# O06 Enhancing antimicrobial stewardship through the SMART-AMS digital model: a strategic framework to tackle antimicrobial resistance

**DOI:** 10.1093/jacamr/dlaf118.006

**Published:** 2025-07-14

**Authors:** Rasha Abdelsalam Elshenawy, Nkiruka Umaru, Zoe Aslanpour

**Affiliations:** Department of Clinical, Pharmaceutical and Biological Sciences, University of Hertfordshire School of Life and Medical Sciences, Hatfield, UK; Department of Clinical, Pharmaceutical and Biological Sciences, University of Hertfordshire School of Life and Medical Sciences, Hatfield, UK; Department of Clinical, Pharmaceutical and Biological Sciences, University of Hertfordshire School of Life and Medical Sciences, Hatfield, UK

## Abstract

**Background:**

Antimicrobial resistance (AMR) is a global health challenge, threatening the effectiveness of antibiotics and infection management [1]. The COVID-19 pandemic exacerbated these issues, disrupting healthcare systems and antimicrobial stewardship (AMS) programmes. AMS is essential for optimizing antimicrobial use, preventing resistance, and safeguarding public health [2].

**Research context:**

This research addressed AMS challenges by implementing a novel framework focused on data-driven strategies to enhance AMS, strengthen healthcare resilience, and combat AMR. Three sequential studies were conducted at a UK NHS Foundation Trust in the East of England. The project was registered under ISRCTN 14825813 and received ethical approval from HRA Ethics and the University of Hertfordshire [3]. Public and patient involvement was ensured through feedback from the Citizens Senate.

**Objectives:**

This research aimed to explore AMS implementation in 2019, before the pandemic, and in 2020 during the COVID-19 pandemic.

**Methods:**

Three studies were undertaken: a systematic literature review evaluating AMS implementation in acute care settings (2000–21), a retrospective analysis of 640 medical records from 2019 and 2020 to assess AMS interventions, and a survey of 240 healthcare professionals to evaluate their AMS knowledge and perceptions. The ‘Start Smart, Then Focus’ approach guided this research.

**Results:**

Significant disruptions in AMS interventions were observed, including reduced antibiotic reviews (81%), audits (70%), and parenteral-to-oral switches (67%). The proposed SMART-AMS model was developed to support proper antibiotic prescribing and AMS practices, integrating innovative tools, such as Machine Learning and Artificial Intelligence, AMS guidance dashboards, AMS pocket guides, adaptive education, and continuous monitoring (Figure 1).

**Conclusions:**

The SMART-AMS model has the potential to promote proper antibiotic prescribing and effective AMS implementation. This model provides a pathway to optimize AMS strategies, safeguard public health, and mitigate the AMR crisis. It enhances AMS globally by supporting precise prescribing and sustainable antibiotic use.

**Figure 1.**
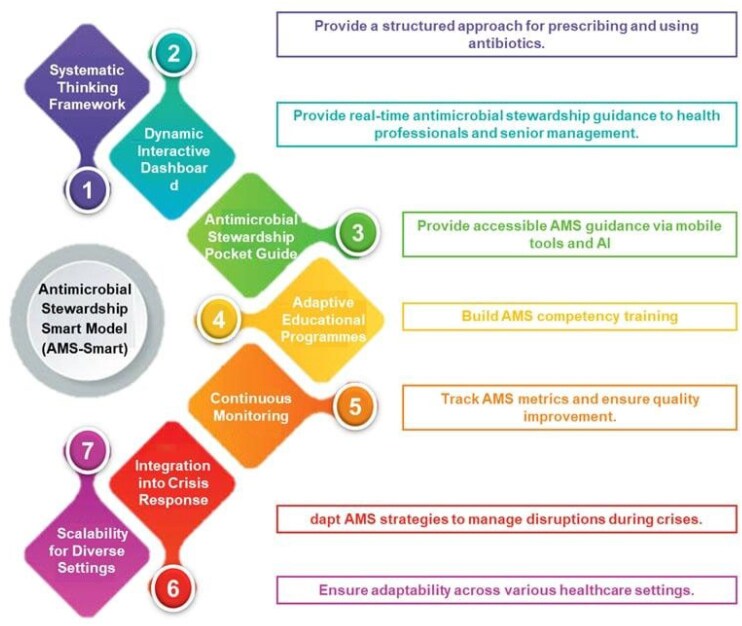
Digital SMART-AMS model components and their key functions.
